# Dapagliflozin stimulates glucagon secretion at high glucose: experiments and mathematical simulations of human A-cells

**DOI:** 10.1038/srep31214

**Published:** 2016-08-18

**Authors:** Morten Gram Pedersen, Ingela Ahlstedt, Mickaël F. El Hachmane, Sven O. Göpel

**Affiliations:** 1 Department of Information Engineering, University of Padua, Italy; 2 AstraZenenca R&D Gothenburg, Dept. CVMD Bioscience, Sweden.

## Abstract

Glucagon is one of the main regulators of blood glucose levels and dysfunctional stimulus secretion coupling in pancreatic A-cells is believed to be an important factor during development of diabetes. However, regulation of glucagon secretion is poorly understood. Recently it has been shown that Na^+^/glucose co-transporter (SGLT) inhibitors used for the treatment of diabetes increase glucagon levels in man. Here, we show experimentally that the SGLT2 inhibitor dapagliflozin increases glucagon secretion at high glucose levels both in human and mouse islets, but has little effect at low glucose concentrations. Because glucagon secretion is regulated by electrical activity we developed a mathematical model of A-cell electrical activity based on published data from human A-cells. With operating SGLT2, simulated glucose application leads to cell depolarization and inactivation of the voltage-gated ion channels carrying the action potential, and hence to reduce action potential height. According to our model, inhibition of SGLT2 reduces glucose-induced depolarization via electrical mechanisms. We suggest that blocking SGLTs partly relieves glucose suppression of glucagon secretion by allowing full-scale action potentials to develop. Based on our simulations we propose that SGLT2 is a glucose sensor and actively contributes to regulation of glucagon levels in humans which has clinical implications.

Insulin and glucagon, released from pancreatic B- and A-cells respectively, are the main regulators of the blood glucose concentration. Insulin is released at high blood glucose levels and directly triggers uptake of the sugar into fat and skeletal muscle tissue and promotes glucose uptake into the liver by stimulating glycogenesis. The significance of loss of B-cell secretory capacity for the development of diabetes is well established and the term islet function is often used synonymously with the ability of B-cells to secrete insulin. However, data demonstrating the contribution of disturbed glucagon secretion to disease development is accumulating. Diabetic subjects suffer from elevated glucagon levels at normal and high glucose concentrations^1^,^2^. Since glucagon triggers glucose release from the liver, too high resting glucagon levels will worsen the situation in diabetic subjects by raising the blood glucose levels even more. In addition, type-1 and type-2 diabetics approaching later stages of the disease often lack the ability to respond to hypoglycemia with increased glucagon secretion, leaving them without protection against potentially life threatening low blood sugar levels^3^,^4^,^5^,^6^,^7^. The point can be made that malfunctioning A-cell stimulus secretion coupling is the driver for hyperglycemia and loss of islet cell function: Upon continuous glucose infusion rats become hyperglycemic after 6 days. This coincides with glucagon hypersecretion whereas insulin release is still unaffected and no signs of insulin resistance are apparent^8^. In addition, B-cell ablation by application of streptocotozin, a widely used diabetes model, does not lead to hyperglycemia in glucagon receptor knock out animals (GlgRKo)^9^. However, streptozotocin treated GlgRKo animals become diabetic when the receptor is re-inserted by adenovirus transfection. Although evidence for the physiological importance of glucagon is constantly increasing, regulation of its release is much less understood than stimulus-secretion coupling in B-cells.

The regulation of glucagon secretion is subject of intense debate and several mechanisms have been proposed. Paracrine regulation by insulin^10^,^11^,^12^,^13^ or zinc^14^ released from B-cells, or by somatostatin released from D-cells^15^, have been proposed to play a dominant role. However, glucagon release is strongly inhibited by glucose concentrations too low to have a substantial effect on insulin secretion^16^ and glucose remains inhibitory on glucagon release even after blockage of somatostatin signaling^16^,^17^. Clearly, glucagon secretion is regulated by several mechanisms but glucose must have a direct effect on A-cells. A-cell inherent glucose sensing has been proposed to depend on store operated channels^18^ or on ATP-sensitive K^+^ (KATP-) channels^19^,^20^. Secretion from A-cells is, as in neuroendocrine cells, triggered by increases in the intracellular Ca^2+^-concentration, which in turn depends on action potential firing. In mouse A-cells Na^+^- and Ca^2+^-current dependent electrical activity is directly regulated by KATP-channel activity^19^,^20^, and we proposed that increasing the glucose concentration leads to closure of KATP-channels, A-cell plasma membrane depolarization and subsequent inactivation of the voltage-dependent Na^+^ and Ca^2+^-channels, thus reducing the amplitude of A-cell action potentials^21^. The inhibitory effect of KATP-channel closure on electrical activity and glucagon secretion was later confirmed in human A-cells^22^. Nevertheless, closure of KATP-channels alone will not depolarize the cell membrane up to the threshold of action potential initiation unless inward background currents are present. Therefore we predicted a none selective background conductance in our mathematical mouse A-cell model^21^.

Recently, SGLT blockers have been developed for the treatment of diabetes. SGLTs are a family of Na^+^/glucose symporters expressed in the kidney, and SGLT blockers reduce blood glucose levels by inhibiting renal glucose reabsorption. SGLTs utilize the electrochemical gradient of sodium to transport glucose into the cell and generate thus an inward current that may lead to depolarization of the cell membrane. It has been demonstrated that two chemically unrelated SGLT2 blockers, dapagliflozin and empagliflozin, despite their glucose lowering effect, increase plasma glucagon levels and raise endogenous glucose production in diabetic subjects^23^,^24^. Further, SGLT2 is expressed in pancreatic A-cells, and its inhibition triggers glucagon release^25^. Here we confirm that dapagliflozin directly affects glucagon secretion and investigate the cellular mechanisms underlying these findings with a mathematical model of A-cell electrical activity developed from human data, potentially explaining the clinical observations in diabetic patients.

## Results

### Glucagon secretion from pancreatic islets

**Figure 1:**
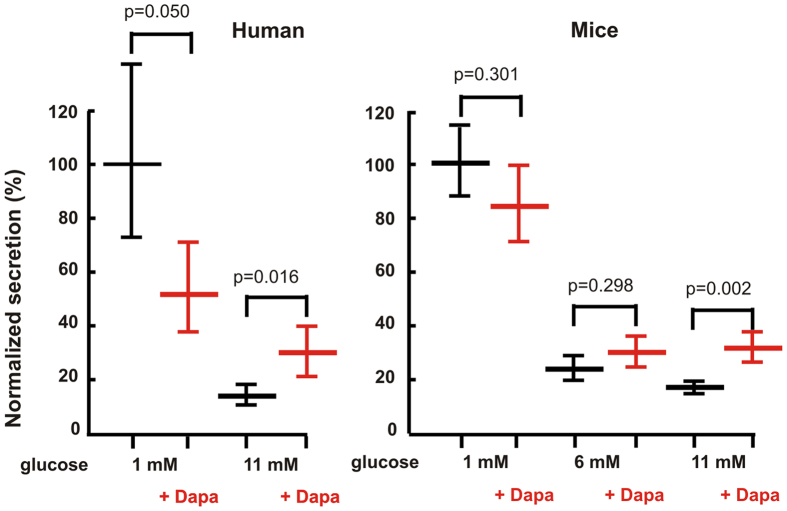
Effect of dapagliflozin (10 nM) on glucagon secretion from human and mouse islets at different glucose concentrations. The figure shows estimates from linear mixed-effects statistical modeling with error bars indicating standard errors. For each islet preparation (i.e., each individual or animal) 6–8 groups of size-matched islets were tested for each indicated condition from 3 donors for human data, and from 29 (1 mM glucose), 11 (6 mM glucose) or 18 (11 mM glucose) animals for mouse data.

Dapagliflozin and empagliflozin, two chemically unrelated SGLT-blockers, both increase plasma glucagon levels in diabetic patients^23^,^24^. We therefore tested the effects of 10 nM dapagliflozin on glucagon secretion in human pancreatic islets (Fig. 1, left). Dapaglifozin is a competitive inhibitor with *in vitro* IC50 value at 2.5 mM glucose of ~1.5 nM, which increases at higher glucose concentrations (unpublished results). A daily dose of 10 mg results in 24–240 nM blood concentration and taking ~93% protein binding into account yields 1.7–17 nM free dapagliflozin^26^. The dose used here thus reflects therapeutic use of the drug.

Raising the glucose concentration from 1 to 11 mM lowered glucagon release to 13% (Fig. 1; p < 0.001 by linear mixed-effects statistical modeling of log-transformed data, see Methods). 10 nM dapagliflozin strongly reduced release-inhibition by 11 mM glucose to 29% of control (p = 0.016), and showed a trend towards an inhibitory effect on secretion at low glucose (51% of control, p = 0.050). Similar results were obtained in mouse islets (Fig. 1, right): at 11 mM glucose, dapagliflozin raised glucagon release from 18% to 32% of control (p = 0.002), while the effect of dapagliflozin addition was minor at 1 mM glucose (84% of control, p = 0.301). At the intermediate glucose concentration of 6 mM, glucagon secretion was estimated to be 24% and 30% of control in the absence or presence of dapagliflozin, respectively (p = 0.298).

### Mathematical modeling

Having established that SGLT2 inhibition stimulates glucagon release at high glucose, we aimed at obtaining mechanistic insight using mathematical modeling. For this purpose, we simulated each individual current for which data from human A-cells were available. Our model thus includes voltage-dependent Na^+^, T-, L- and P/Q-type Ca^2+^, as well as A-type and delayed-rectifier K^+^ channels, in addition to KATP channels. Parameters where chosen to generate currents as close to experimental data^22^ as possible, and then adjusted minimally in order to obtain stable electrical activity in the whole-cell model. Since A-cells express SGLTs^25^, we also added a mathematical model of SGLT2 co-transport and the related electrogenic current, based on the model originally formulated for rabbit SGLT1 by Parent *et al*.^27^, and subsequent modifications to account for differences between rabbit and humans^28^, and between SGLT1 and SGLT2^29^.

The SGLT2 current carried by Na^+^ co-transport is a depolarizing current, as is the SGLT1 current responsible for initiating electrical activity and GLP-1 release from intestinal L-cells^30^,^31^. It is thus counterintuitive how the depolarizing SGLT2 current can lower glucagon secretion from A-cells. However, blocking the hyperpolarizing current through KATP channels with tolbutamide also inhibits glucagon release both in mouse^19^ and human^22^ islets, in contrast to its stimulating effect on insulin release. The particular properties of A-cell electrophysiology have been suggested to underlie the inhibitory effect of tolbutamide in A-cells, where tolbutamide application depolarizes the cell but lowers action potential amplitude^19^,^20^,^32^. Mathematical modeling has shown that the electrophysiological properties of mouse A-cells can account for the reduction of action potential amplitude in response to KATP channel closure^21^,^33^, and that this leads to inhibition of glucagon secretion due to reduced open probability of P/Q-type Ca^2+^ channels^34^.

**Figure 2:**
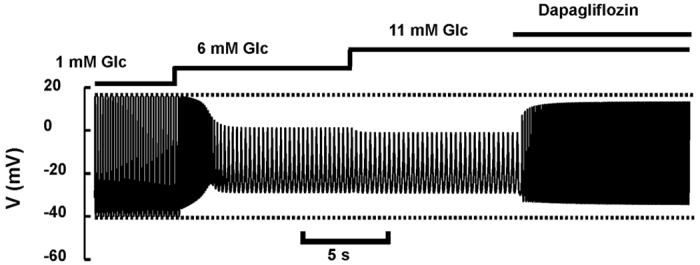
Model simulation of electrical activity. The glucose concentration was raised from 1 mM to 6 mM and 11 mM, followed by simulated dapagliflozin application, as indicated. Simulations were performed by changing the glucose parameter in the SGLT2 submodel, G_SGLT2_, from 1 mM to 6 mM to 11 mM, and by lowering the KATP conductance from 0.15 to 0.115 nS to model the increase in glucose concentration to 6 or 11 mM, since inhibition of KATP channel activity is maximal already at 6 mM glucose^31^. Dapagliflozin application was assumed to block all SGLT2 transporters, which was modeled by setting the parameter *n* = 0.

Therefore, we speculated that in pancreatic A-cells SGLT2 activation might have an effect similar to KATP channel blockage. Our simulations predict that AP firing is reduced in frequency and amplitude when the external glucose concentration is increased from 1 to 6 or 11 mM (Fig. 2), in agreement with membrane potential recordings from mouse A-cells^32^. Simulating addition of dapagliflozin in the presence of high glucose reverts action potential height and frequency almost back to what was observed at low glucose alone. Since glucagon secretion is highly dependent on the action potential height^22^,^34^,^35^, this simulation suggests that SGLT2 blockers stimulate glucagon secretion at high glucose concentrations by allowing close-to-complete action potentials to develop. The AP amplitude is slightly (~3 mV) higher at 6 mM than at 11 mM glucose in our simulation (Fig. 2), which is entirely due to an effect of glucose on SGLT2 in the model, suggesting that SGLT2 can act as a glucose sensor in A-cells. This assumption corresponds to the findings that whereas KATP channel closure is saturated at 6 mM^32^, SGLT2 currents are not ref. 36.

### Negligible reduction in cellular glucose uptake cannot explain dapagliflozin-induced stimulation of secretion

In the simulation in Fig. 2 we assumed that glucose acts in two ways on the A-cell action potential: via stimulation of the SGLT2 current (modeled as an increase of the parameter G_SGLT2_ in the model), and via glucose metabolism, ATP production and closure of KATP-channels (modeled as a decrease of the parameter g_KATP_ in the model). Dapagliflozin application was assumed to remove the SGLT2 current without affecting the KATP conductance. However, it has been suggested that the stimulatory effect of dapagliflozin is a result of reduced glucose influx and metabolism when SGLT2-mediated glucose transport is blocked, which was speculated to open KATP channels and allow full action potentials to develop^25^.

We investigated theoretically whether the effect of dapagliflozin on glucose metabolism is feasible. Heimberg *et al*.^37^ found that, at 10 mM glucose, glucose enters islet non-B-cells (mostly A-cells) at a rate of approximately 50 μM/s via GLUT1 (50 μmol per liter of cell volume). Glucose utilization was measured to be ~5 μM/s. In steady state, the net influx balances consumptions, and thus GLUT1 must transport glucose outward at ~45 μM/s, effectively equilibrating extra- and intra-cellular glucose levels^38^. The SGLT2-mediated glucose fluxes predicted by our model are, based on a cell volume of 0.8 pL^39^,^40^, 0.8 and 1.7 μM/s at 1 and 11 mM glucose, respectively. Thus, GLUT1 transports glucose with rate at least an order of magnitude greater than our estimate of SGLT2-mediated uptake. Glucose uptake via GLUT1 is driven by the glucose concentration gradient across the membrane and is not the rate limiting step in A-cell glucose metabolism whereas glucose transport via SGLT2 is driven by the Na^+^ gradient across the cell membrane. Reduction in glucose inflow via SGLT2 will thus to a large extent be compensated for by an increased glucose net-inflow via GLUT1 (due to reduced outflow) as summarized by the steady-state relation *J*_in_ = (*k*_out_ + *k*_consumption_) × *G*_i_.

As a consequence the ~3% reduction in glucose influx due to SGLT2 blockage will lower intracellular glucose levels (*G*_i_) by a similar fraction, and therefore glucose consumption will decrease by only ~3%. Modest nonlinearities in glucose consumption such as for glucokinase^38^ will not change this conclusion. It is therefore highly unlikely that the reduction in glucose uptake due to SGLT2 inhibition reduces glucose metabolism and affects KATP-channel activity substantially. We therefore propose that SGLT2 inhibitors augment glucagon secretion principally because of direct effects on the membrane potential via interference with electrogenic Na^+^/glucose transport. Similar conclusions have been drawn from direct measurements of intracellular glucose in intestinal L-cells^30^. Finally, we note that our estimate of glucose uptake via SGLT2 is constrained by the electrophysiology in our model. If we increase the number of SGLT2 transporters in the model 5–10 fold to make SGLT2-mediated glucose uptake more comparable to GLUT1 transport, the model no longer produces action potentials resembling experimental recordings^22^ (Supplementary Fig. S4).

### SGLT2 currents contribute to shape action potentials

**Figure 3:**
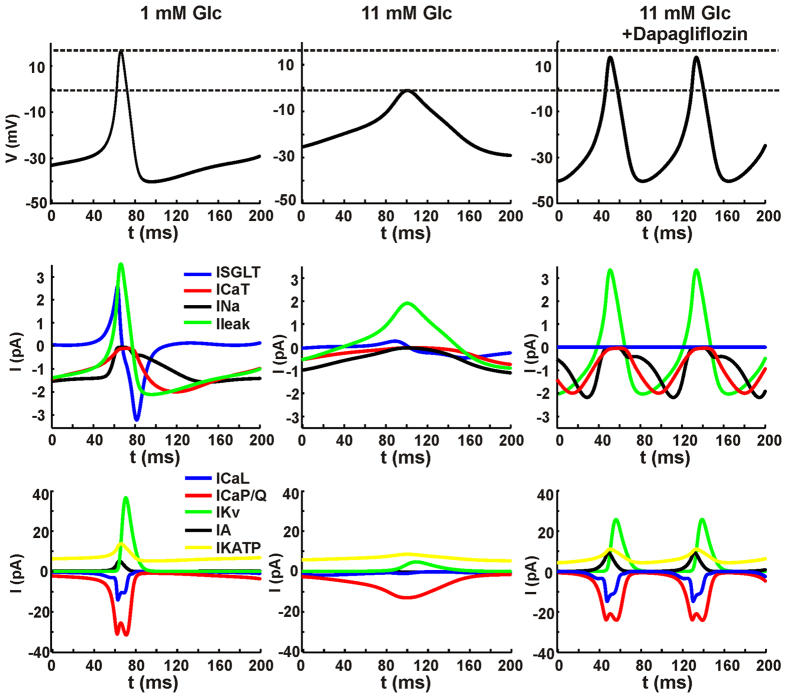
Ion currents during simulated action potentials during low glucose, high glucose, and high glucose in the presence of dapagliflozin. The upper panels show zooms on action potentials under the various conditions as indicated. The middle and lower panels show the simulated ion currents during the action potentials, as indicated in the legend. Note the different scales on the y-axes of the middle and lower panels.

Having clarified that dapagliflozin stimulates glucagon secretion at high glucose via a direct effect on action potential height, we investigated how SGLT2 currents contribute to shaping the A-cell action potential in our model. In response to depolarizations, the SGLT2 current exhibits both a transient, outward component, and a sustained, inward component^29^. These components are also seen in our simulations of human A-cell electrical activity (Fig. 3).

At low glucose levels, action potentials are initiated when inward Na^+^ and Ca^2+^ currents depolarize the cell sufficiently to open high-voltage activated (HVA) L- and P/Q-type Ca^2+^ channels, which drive the upstroke of the action potential. During the upstroke, NaV channels inactivate, A-type K^+^ channel activate, and the transient, outward component of the SGLT2 current is evident. These events limit the height of the action potential, which is terminated when delayed-rectifier K^+^ currents activate. During the downstroke, the transient component of the SGLT2 current is inward and acts as a brake. The membrane potential drops to −40 mV, which allows reactivation of HVA Ca^2+^ channels, thus preparing the cell for the creation of the next action potential. Between action potentials, the SGLT2 current is slightly positive (outward).

At high glucose, or during tolbutamide application, the reduction in KATP channel activity has a number of effects. Most importantly, after an action potential, there is insufficient outward current to repolarize the membrane potential sufficiently to reactivate Na^+^ and Ca^2+^ channels. The transient inward SGLT2 current acting as a brake during the downstroke aggravates the situation. Further, the sustained, inward component of the SGLT2 current is more important than at low glucose concentration. As a result, the SGLT2 current is now inward between action potentials. Dapagliflozin application removes both the transient and sustained components of the SGLT2 current with converging effects. The removal of the sustained, inward component has the same effect as increasing the KATP-channel conductance. The lack of the transient, inward current permits K^+^ channels to repolarize the membrane potential more after an action potential. Both mechanisms allow reactivation of Na^+^ and Ca^2+^ channels and almost full action potential generation in the presence of dapagliflozin.

## Discussion

Here we confirm clinical findings showing that SGLT2 inhibitors increase glucagon secretion at elevated glucose levels, and based on our theoretical investigations we propose that dapagliflozin activate pancreatic A-cells mainly via effects on electrical activity. In particular, we show that inhibition of SGLT2 with dapagliflozin doubles glucagon secretion at high glucose concentrations (Fig. 1).

We calculated that SGLT2-mediated glucose transport contributes to only ~3% of total glucose uptake in A-cells. This result indicates that inhibition of SGLT2 leads to minimal changes in glucose metabolism and KATP-closure, and rather suggests that it is the removal of the SGLT2-associated electrical currents that influence electrical activity. Closure of KATP-channels reduces action potential height in mouse and human A-cells^19^,^22^,^35^, which we attribute to voltage-dependent inactivation of the Na^+^ and Ca^2+^ channels carrying the action potentials in this cell type. Our mathematical model of human A-cell electrophysiology predicts that SGLT2 inhibition can allow full-scale action potentials to develop when KATP-channel activity is reduced, i.e., at high glucose concentrations (Fig. 2). Since glucagon secretion depends strongly on the height of action potentials both in mice and human A-cells^22^,^35^, we suggest that the effects of SGLT2 inhibition on action potential height lie at the heart of dapagliflozin-stimulated glucagon secretion.

Our model simulations suggest that SGLT2 might be directly involved in glucose sensing in A-cells. Without changing the KATP conductance, an increase in the glucose concentration sensed by SGLT2 from 6 mM to 11 mM results in a small reduction of AP height (Fig. 2). With other model parameters, which could represent a subset of A-cells, the simulated effect can be more pronounced, and AP may be present at 6 mM but fail to develop at 11 mM glucose (Supplementary Fig. 7), entirely because of SGLT2 glucose sensing. The further reduction of simulated AP height when increasing glucose levels from 6 to 11 mM may also underlie the fact that 6 mM glucose inhibited glucagon secretion slightly less than 11 mM in our experiments on mouse islets (~24% vs. ~18% of control, Fig. 1). In the presence of dapagliflozin, SGLT2 can no longer act as a glucose sensor, and since KATP-conductance was assumed to be identical at 6 and 11 mM glucose, the model predicts that secretion is identical at 6 and 11 mM glucose in the presence of dapagliflozin, again in agreement with the experimental data (~32% vs. 30%, Fig. 1). In other words, the lack of a statistically significant effect of dapagliflozin at 6 mM glucose may be attributed to insufficient activation of the SGLT2-dependent glucose sensing system.

**Figure 4:**
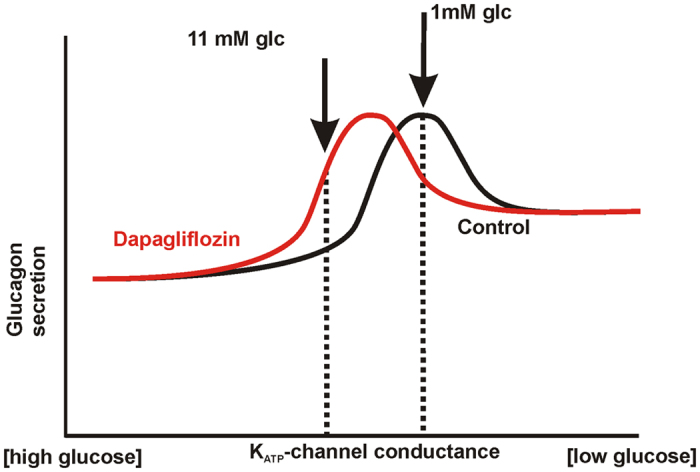
Schematic representation of the control of glucagon secretion by KATP-conductance and SGLT2.

The SGLT2-associated current consists of two components, a transient and a sustained^29^. We find that the transient component acts a break during the downstroke of the action potentials (Fig. 3). The sustained, inward component tends to depolarize the cell, similarly to KATP-channel closure. Both components will tend to prevent repolarization and reactivation of Na^+^ and Ca^2+^ channels needed for full action potential generation, thus reducing the height of the action potentials. Removal of these inhibiting actions of the SGLT2-current by pharmacological inhibition will have the same effect as KATP-channel opening: reactivation of voltage-inactivated channels and increased action potential height (Fig. 4).

In human A-cells the resting conductance was ~120 pS no matter if the A-cells were exposed to 1 or 6–10 mM glucose, and addition of tolbutamide lead to a small decrease in resting conductance that did not reach significance^22^. This background conductance corresponds to just 6 open KATP-channels on average^41^. The failure of tolbutamide to further decrease the resting conductance indicates that other background conductances must be present. In rodent A-cells the resting conductance appears to be at least twice as large as in human A-cells and glucose-induced reduction of the resting conductance becomes detectable: Raising the glucose concentration from 1 to 6 mM reduced the resting conductance from 270 pS by 75 pS^32^ or ~25%. Almost the same reduction was obtained by adding tolbutamide in the presence of 1 mM glucose, whereas adding the drug in the presence of 6 mM had no further effect. Again, this indicates the presence of another, so far unknown background conductance. In contrast to KATP-channels, SGLT2 is activated when the glucose concentration is increased and will thus partly obscure the glucose induced reduction in KATP-channel conductance, explaining the extremely small and in case of human A-cells, absence of glucose-induced detectable changes in resting conductance. In our model, the SGLT2 mediated component of the background conductance, calculated from simulated 10 mV depolarizations from −70 mV, increases by ~7 pS (Supplementary Fig. 6) when glucose increases from 1 to 11 mM, an amount comparable to the average reduction in background conductance in response to tolbutamide^22^. Partial glucose sensing via SGLT2 thus fits well to the reported tiny or absent changes in background conductance upon glucose stimulation. Nevertheless, A-cells possess significant “spare” KATP-channel conductance as evidenced by diazoxide’s ability to overcome glucose-induced suppression of secretion. In mice A-cells 100 μM diazoxide increases KATP-channel conductance to more than 2 nS compared to 0.27 nS observed at 1 mM glucose where maximal secretion is obtained without pharmacological interference^32^. Contribution of SGLT2 to glucose sensing can thus explain how glucose can change the resting potential with changes in background conductance at or below the detection level. It would also be compatible with the observation that diazoxide can override glucose inhibition of glucagon secretion by opening of this spare KATP-conductance.

Overall, according to our model glucose dependence of glucagon secretion would mainly depend on the balance of background currents of which so far KATP-channels and now SGLT2 have been identified. Potentially other unidentified currents contribute in setting the resting membrane potential. Reported results on glucagon secretion vary considerably. For example sulfonylureas have been reported to increase, decrease and to have no effect on secretion^15^,^42^,^43^,^44^. Also results with the KATP-channel opener diazoxide vary, and the stimulatory effect of low dose diazoxide on secretion in the presence of high glucose could not be reproduced in a study by Cheng-Zue *et al*.^15^. Some of the reported differences are most likely due to experimental differences (static incubation vs perifusion, presence of amino acids, different animal strains etc.). However our model would offer explanation to some of the divergences: A bell shaped KATP-channel dependent secretion implies that the effect of KATP-channel modulators strongly depends on the metabolic state of the A-cell. In addition, ATP/ADP ratio sensing of the A-cell is complex and might differ between preparations. Although A- and B-cells express exactly the same KATP-channel subunits the ATP sensitivity is ~3 times higher in A-cells and dynamically regulated by insulin^45^ contributing to the high experimental variation. As a result, adding the same concentration of diazoxide might either increase or decrease the secretion response dependent on where the cells are on the bell shaped curve (Fig. 4).

Since the input resistance of the A-cell is very high, meaning the resting potential is set by very few open channels, already small changes in ion channel expression will have major effects, contributing to the variation seen in secretion measurements. Recently Bonner *et al*.^25^ demonstrated that progression towards diabetes in human and mice as well as culturing islets in high glucose for 24 h strongly increases SGLT2 expression. Apparently SGLT2 expression is under dynamic control, which will change the balance of the currents setting the resting membrane potential and contribute to the observed experimental variation. In the same study glucagon secretion was measured with similar results to ours, the main difference being that dapagliflozin amplified hormone release also at 1 mM glucose. This might be due to experimental variations as outlined above and possibly due to the high dapagliflozin concentration (12 μM) used compared to our study (10 nM).

### Clinical implications

Too high basal glucagon levels and the lost ability to respond to hypoglycemia with sufficient glucagon release are contributing factors to the diabetic phenotype^2^. Therefore, the potentiating effects of SGLT2 inhibition on glucagon secretion at elevated glucose levels might be viewed as problematic. Indeed, both dapagliflozin and empagliflozin, two chemically unrelated SGLT blockers, raise basal glucagon levels and hepatic glucose output. Even though it has not been demonstrated directly, it is fair to assume that the elevated glucagon levels cause the latter. However, since dapagliflozin lowers blood sugar levels via urinary glucose disposal, simultaneous increased hepatic glucose output might even improve total glucose disposal and by that potentially induce weight loss.

According to our model glucagon secretion occurs in a certain window of resting potential and requires thus the right balance between KATP-channel and SGLT2 activity. Too strong depolarization will turn of secretion via inactivation of voltage-gated Na^+^ and Ca^2+^ channels, whereas strong hyperpolarization reduces secretion since the threshold for action potential generation will not be reached. Inhibiting SGLTs blocks the entry of positive Na^+^-ions through the transporter and has thus a hyperpolarizing effect and could therefore potentially compromise glucagon secretion at low blood sugar levels. We found that glucagon secretion was virtually unaffected in mouse islets at 1 mM glucose, whereas there was a tendency towards lower secretion in the more limited human dataset. In contrast, Bonner *et al*.^25^ found that glucagon secretion was augmented by dapagliflozin at low glucose concentrations in human islets. Further studies are needed to clarify these aspects. Nonetheless, SGLT2 inhibition has only minor if any effect on the risk of hypoglycemia in clinical findings^46^,^47^,^48^.

## Material and Methods

### Mice islet preperation and secretion measurements

Mice (C57black 12–20 weeks) were anesthetized (isoflurane) and killed by cervical dislocation. 0.9 U/ml collagenase P (cat no:1 213 873, Roche) was injected into the pancreatic duct, pancreata were removed and digested with collagenase for 17 min at 37 °C after which the samples were vigorously shaken. The digest was transferred into ice cold Hanks buffer with Ca^2+^ and Mg^2+^ and NaHCO_3_ (cat no 14025, Gibco), washed and islets hand-picked into RPMI-1640 media with Glutamax (Gibco 72400-021), 10% FCS and 1% PEST at 37 °C and 5% CO2 and cultured for 48–72 hours before use. For glucagon secretion measurements, islets were washed twice in KRH buffer supplemented with 5.6 mM glucose and 0.1% BSA in petridishes and then preincubated in KRH 5.6 mM glucose and 0.1% BSA for 30 min at 37 °C, 5% CO_2_. After preincubation, islets were washed twice with 5.6 mM glucose in KRH and 0.1% BSA. 3 size matched islets/tube were incubated for 1 h at 37 °C in (KRH) supplemented with indicated constituents. After incubation 100 μl/tube supernatant was harvested into a low binding eppendorf tube and frozen at −80 °C. Glucagon was analyzed using a glucagon ELISA kit (Mercodia, Uppsala, Sweden). For each islet preparation 6–8 tubes were incubated under the same condition. Experimental procedures were approved by the Local Ethics Review Committee on Animal Experiments (Göteborg Region, Sweden) and all experiments were carried out in accordance with all relevant guidelines.

### Human islet preparation and secretion measurements

Primary human islets were received from Prodo Laboratories Inc (Irvine, CA, USA) and kept in Prodo Islet Media Standard ((PIM(S))complete. For secretion measurements, islets were washed twice in 5.6 mM glucose, 0.1% BSA, KRH buffer. 3 islets/tube were incubated for 1 h at 37 °C in KRH buffer supplemented with indicated constituents and glucagon secretion was measured after 1 h incubation. Glucagon ELISA (Mercodia AB, Uppsala, Sweden), was run according to manufacturer’s protocol. For each islet preparation 6–8 tubes were incubated under the same condition.

### Insphero islets

Re-aggregated human islets where ordered from Insphero and used according to the manufacturer’s instructions. In short, one re-aggregated islet/well was cultured in GravtiyTRAP^TM^ wells in Islet Microtissue Maintenance Medium until use. For glucagon secretion measurement islets were incubated for 2 h at the indicated glucose concentrations. Glucagon ELISA (Mercodia AB, Uppsala, Sweden), was run according to manufacturer’s protocol. For each islet preparation 6–8 wells were incubated under the same condition.

### Mathematical modeling

A mathematical Hodgkin-Huxley-type model that describes electrical activity in human pancreatic A-cells was developed based on patch clamp data from human pancreatic A-cells^22^. The model includes ATP-sensitive K^+^ channels (KATP-channels), a passive leak current, voltage-gated Na^+^-, K^+^- and Ca^2+^-channels, and the electrogenic sodium glucose co-transporter SGLT2.

The evolution of the membrane potential V is driven by the contribution from the different currents,


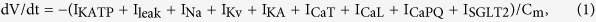


where C_m_ is the cell membrane capacitance. Voltage-gated membrane currents are modeled as


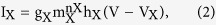


where X stands for the channel type, V_X_ is the associated reversal potential, g_X_ the maximal whole-cell channel conductance, and m_X_ and h_X_ describe activation and inactivation of the channel, respectively.

Activation (similarly inactivation) is described by


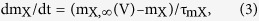


where m_X,∞_(V) is the steady-state voltage-dependent activation function, and τ_mX_ is the time-constant of activation, which in some cases depends on the membrane potential.

Steady-state voltage-dependent activation (inactivation) functions were described by the Boltzmann equation


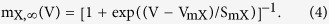


SGLT2 was modeled as a six-state model with the associated current related to movements of charged residues of the transporters as in the SGLT1 model of Parent *et al*.^27^, with modification to account for species differences^28^, and differences between SGLT1 and SGLT2^29^.

Details of the individual currents and parameters can be found in the Supplementary Material. Simulations were performed in MATLAB™ (R2014a, MathWorks Inc., Natick, MA, USA) using the ode15s solver. Computer code is available as Supplementary Material.

### Statistics

Glucagon secretion measurements were highly non-normal distributed and were therefore log-transformed to improve normality. The log-transformed data were fitted to a linear statistical mixed-effects model with low/high glucose and absence/presence of dapagliflozin as covariates including an interaction term (fixed effects) and individual/animal as random effect to take into account the fact that measurements performed on islet preparations from the same individual/animal are correlated. P-values were calculated from two-sided t-tests, and considered significant at the 0.05 level. All analyses were performed in R version 3.0.2^49^ using the lm and lme (from the nlme package^50^) functions as appropriate.

## Additional Information

**How to cite this article**: Pedersen, M. G. *et al*. Dapagliflozin stimulates glucagon secretion at high glucose: experiments and mathematical simulations of human A-cells. *Sci. Rep.* **6**, 31214; doi: 10.1038/srep31214 (2016).

## Supplementary Material

Supplementary Information

Supplementary Information

## References

[b1] Baron A. D., Schaeffer L., Shragg P. & Kolterman O. G. Role of Hyperglucagonemia in Maintenance of Increased Rates of Hepatic Glucose Output in Type II Diabetics. Diabetes 36, 274–283 (1987). 2879757 10.2337/diab.36.3.274

[b2] D’Alessio D. The role of dysregulated glucagon secretion in type 2 diabetes. Diabetes, Obesity and Metabolism 13, 126–132 (2011). 10.1111/j.1463-1326.2011.01449.x 21824266

[b3] Segel S. A., Paramore D. S. & Cryer P. E. Hypoglycemia-Associated Autonomic Failure in Advanced Type 2 Diabetes. Diabetes 51, 724–733 (2002). 11872673 10.2337/diabetes.51.3.724

[b4] Cryer P. E. Hypoglycaemia: The limiting factor in the glycaemic management of type I and type II diabetes. Diabetologia 45, 937–948 (2002). 12136392 10.1007/s00125-002-0822-9

[b5] Bolli G., Tsalikian E., Haymond M., Cryer P. E. & Gerich J. E. Defective glucose couterregulation after subcutaneous insulin in nonsinslin-depedent diabeted mellitus. Paradoxical suppression of glucose utilization and lack of compensatory increase in glucose production, roles of insulin resistance, abnormal neuroendocrine responses, and islet paracrine interactions. J. Clin. Invest. 73, 1532–1541 (1984). 6373827 10.1172/JCI111359 PMC437063

[b6] Meneilly G. S., Cheung E. & Tuokko H. Counterregulatory Hormone Responses to Hypoglycemia in the Elderly Patient with Diabetes. Diabetes 43, 403–410 (1994). 8314012 10.2337/diab.43.3.403

[b7] Shamoon H. . Increased epinephrine and skeletal muscle responses to hypoglycemia in non-insulin-dependent diabetes mellitus. J. Clin. Invest. 93, 2562–2571 (1994). 8200993 10.1172/JCI117267 PMC294484

[b8] Jamison R. A. . Hyperglucagonemia precedes a decline in insulin secretion and causes hyperglycemia in chronically glucose-infused rats. American Journal of Physiology - Endocrinology and Metabolism 301, E1174–E1183 (2011). 21862723 10.1152/ajpendo.00175.2011 PMC3233775

[b9] Lee Y. . Metabolic manifestations of insulin deficiency do not occur without glucagon action. Proceedings of the National Academy of Sciences 109, 14972–14976 (2012). 10.1073/pnas.1205983109 PMC3443167 22891336

[b10] Buchanan K. D. & Mawhinney W. A. A. Insulin Control of Glucagon Release From Insulin-deficient Rat Islets. Diabetes 22, 801–803 (1973). 4270691 10.2337/diab.22.11.801

[b11] Greenbaum C. J., Havel P. J., Taborsky J. & Klaff L. J. Intra-islet insulin permits glucose to directly suppress pancreatic A-cell function. J. Clin. Pharmacol. 88, 767–773 (1991). 10.1172/JCI115375 PMC295460 1679440

[b12] Muller W. A., Faloona G. R. & Unger R. H. The effect of experimental insulin deficiency on glucagon secretion. J. Clin. Invest. 50, 1992–1999 (1971). 4935445 10.1172/JCI106691 PMC292125

[b13] Maruyama H., Hisatomi A., Orci L., Grodsky G. & Unger R. Insulin within islets is a physiologic glucagon release inhibitor. J. Clin. Invest. 74, 2296–2299 (1984). 6392344 10.1172/JCI111658 PMC425424

[b14] Ishihara H., Maechler P., Gjinovci A., Herrera P.-L. & Wollheim C. B. Islet [beta]-cell secretion determines glucagon release from neighbouring [alpha]-cells. Nat Cell Biol 5, 330–335 (2003). 12640462 10.1038/ncb951

[b15] Cheng-Xue R. . Tolbutamide Controls Glucagon Release From Mouse Islets Differently Than Glucose: Involvement of KATP Channels From Both α-Cells and δ-Cells. Diabetes 62, 1612–1622 (2013). 23382449 10.2337/db12-0347 PMC3636641

[b16] Vieira E., Salehi A. & Gylfe E. Glucose inhibits glucagon secretion by a direct effect on mouse pancreatic alpha cells. Diabetologia 50, 370–379 (2007). 17136393 10.1007/s00125-006-0511-1

[b17] de Heer J., Rasmussen C., Coy D. H. & Holst J. J. Glucagon-like peptide-1, but not glucose-dependent insulinotropic peptide, inhibits glucagon secretion via somatostatin (receptor subtype 2) in the perfused rat pancreas. Diabetologia 51, 2263–2270 (2008). 18795252 10.1007/s00125-008-1149-y

[b18] Liu Y.-J., Vieira E. & Gylfe E. A store-operated mechanism determines the activity of the electrically excitable glucagon-secreting pancreatic [alpha]-cell. Cell Calcium 35, 357–365 (2004). 15036952 10.1016/j.ceca.2003.10.002

[b19] Göpel S. O. . Regulation of glucagon release in mouse alpha-cells by KATP channels and inactivation of TTX-sensitive Na^+^ channels. *J Physiol* (*Lond*) 528, 509–520 (2000). 11060128 10.1111/j.1469-7793.2000.00509.x PMC2270147

[b20] MacDonald P. . A K ATP channel-dependent pathway within alpha cells regulates glucagon release from both rodent and human islets of Langerhans. Plos Biol 5, 1236–1247 (2007). 10.1371/journal.pbio.0050143 PMC1868042 17503968

[b21] Diderichsen P. & Göpel S. O. Modelling the electrical activity of pancreatic A-cells based on experimental data from intact mouse islest. J. Biol. Physics 32, 209–229 (2006). 10.1007/s10867-006-9013-0 PMC2651523 19669464

[b22] Ramracheya R. . Membrane Potential-Dependent Inactivation of Voltage-Gated Ion Channels in α-Cells Inhibits Glucagon Secretion From Human Islets. Diabetes 59, 2198–2208 (2010). 20547976 10.2337/db09-1505 PMC2927942

[b23] Merovci A. . Dapagliflozin improves muscle insulin sensitivity but enhances endogenous glucose production. The Journal of Clinical Investigation 124, 509–514 (2014). 24463448 10.1172/JCI70704 PMC3904617

[b24] Ferrannini E. . Metabolic response to sodium-glucose cotransporter 2 inhibition in type 2 diabetic patients. J. Clin. Inv. 124, 499–508 (2014). 10.1172/JCI72227 PMC3904627 24463454

[b25] Bonner C. . Inhibition of the glucose transporter SGLT2 with dapagliflozin in pancreatic alpha cells triggers glucagon secretion. Nat Med 21, 512–517 (2015). 25894829 10.1038/nm.3828

[b26] Kasichayanula S. . Pharmacokinetics and pharmacodynamics of dapagliflozin, a novel selective inhibitor of sodium–glucose co-transporter type 2, in Japanese subjects without and with type 2 diabetes mellitus. Diabetes, Obesity and Metabolism 13, 357–365 (2011). 10.1111/j.1463-1326.2011.01359.x 21226818

[b27] Parent L., Supplisson S., Loo D. & Wright E. M. Electrogenic properties of the cloned Na^+^/glucose cotransporter: I. Voltage-clamp studies. J. Membr. Biol. 125, 49–62 (1992). 1542106 10.1007/BF00235797

[b28] Hirayama B. A. . Kinetic and specificity differences between rat, human, and rabbit Na^+^-gllucose cotransporters (SGLT-1). Am. J. Physiol. 270, G919–926 (1996). 8764197 10.1152/ajpgi.1996.270.6.G919

[b29] Mackenzie B., Loo D. D. F., Panayotova-Heiermann M. & Wright E. M. Biophysical characteristics of the pig kidney Na^+^/glucose cotransporter SGLT2 reveal a common mechanism for SGLT1 and SGLT2. J. Biol. Chem. 271, 32678–32683 (1996). 8955098 10.1074/jbc.271.51.32678

[b30] Parker H. E. . Predominant role of active versus facilitative glucose transport for glucagon-like peptide-1 secretion. Diabetologia 55, 2445–2455 (2012). 22638549 10.1007/s00125-012-2585-2 PMC3411305

[b31] Gribble F. M., Williams L., Simpson A. K. & Reimann F. A Novel Glucose-Sensing Mechanism Contributing to Glucagon-Like Peptide-1 Secretion From the GLUTag Cell Line. Diabetes 52, 1147–1154 (2003). 12716745 10.2337/diabetes.52.5.1147

[b32] Zhang Q. . Role K_ATP_ channels in glucose-regulated glucagon secretion and impaired counterregulation in Type 2 diabetes. Cell Metabolism 18, 871–882 (2013). 24315372 10.1016/j.cmet.2013.10.014 PMC3851686

[b33] Watts M. & Sherman A. Modeling the Pancreatic α-Cell: Dual Mechanisms of Glucose Suppression of Glucagon Secretion. Biophysical Journal 106, 741–751 (2014). 24507615 10.1016/j.bpj.2013.11.4504 PMC3944880

[b34] Montefusco F. & Pedersen M. G. Mathematical modelling of local calcium and regulated exocytosis during inhibition and stimulation of glucagon secretion from pancreatic alpha-cells. The Journal of Physiology 593, 4519–4530 (2015). 26236035 10.1113/JP270777 PMC4606540

[b35] Göpel S. . Capacitance measurements of exocytosis in mouse pancreatic {alpha}-, {beta}- and {delta}-cells within intact islets of Langerhans. *J Physiol* (*Lond*) 556, 711–726 (2004). 14966302 10.1113/jphysiol.2003.059675 PMC1664984

[b36] Hummel C. S. . Glucose transport by human renal Na^+^/d-glucose cotransporters SGLT1 and SGLT2. American Journal of Physiology - Cell Physiology 300, C14–C21 (2011). 20980548 10.1152/ajpcell.00388.2010 PMC3023189

[b37] Heimberg H., De Vos A., Pipeleers D., Thorens B. & Schuit F. Differences in Glucose Transporter Gene Expression between Rat Pancreatic α- and β-Cells Are Correlated to Differences in Glucose Transport but Not in Glucose Utilization. Journal of Biological Chemistry 270, 8971–8975 (1995). 7721807 10.1074/jbc.270.15.8971

[b38] Sweet I. R. & Matschinsky F. M. Are there kinetic advantages of GLUT2 in pancreatic glucose sensing? Diabetologia 40, 112–119 (1997). 9028727 10.1007/s001250050652

[b39] Barg S., Galvanovskis J., Göpel S. O., Rorsman P. & Eliasson L. Tight coupling between electrical activity and exocytosis in mouse glucagon-secreting alpha-cells. Diabetes 49, 1500–1510 (2000). 10969834 10.2337/diabetes.49.9.1500

[b40] Göpel S., Kanno T., Barg S., Galvanovskis J. & Rorsman P. Voltage-gated and resting membrane currents recorded from B-cells in intact mouse pancreatic islets. The Journal of Physiology 521, 717–728 (1999). 10601501 10.1111/j.1469-7793.1999.00717.x PMC2269694

[b41] Arkhammar P., Nilsson T., Rorsman P. & Berggren P. O. Inhibition of ATP-regulated K^+^ channels precedes depolarization-induced increase in cytoplasmic free Ca^2+^ concentration in pancreatic beta-cells. Journal of Biological Chemistry 262, 5448–5454 (1987). 2437108

[b42] Bohannon N. V., Lorenzi M., Grodsky G. M. & Karam J. H. Stimulatory Effects of Tolbutamide Infusion on Plasma Glucagon in Insulin-Dependent Diabetic Subjects. Journal of Clinical Endocrinology & Metabolism 54, 459–462 (1982). 7054228 10.1210/jcem-54-2-459

[b43] Silvestre R. A., Miralles P., Moreno P., Villanueva M. L. & Marco J. Somatostatin, insulin and glucagon secretion by the perfused pancreas from the cysteamine-treated rat. Biochemical and Biophysical Research Communications 134, 1291–1297 (1986). 2868720 10.1016/0006-291x(86)90390-6

[b44] Loreti L., Sugase T. & Foa P. P. Diurnal variations of serum insulin, total glucagon, cortisol, glucose and free fatty acids in normal and diabetic subjects before and after treatment with chlorpropamide. Hormone Res. 5, 278–292 (1974). 4851417 10.1159/000178641

[b45] Leung Y. M. . Insulin Regulates Islet α-Cell Function by Reducing KATP Channel Sensitivity to Adenosine 5′-Triphosphate Inhibition. Endocrinology 147, 2155–2162 (2006). 16455778 10.1210/en.2005-1249

[b46] Bailey C. J. . Efficacy and safety of dapagliflozin monotherapy in people with Type 2 diabetes: a randomized double-blind placebo-controlled 102-week trial. Diabetic Medicine 32, 531–541 (2015). 25381876 10.1111/dme.12624

[b47] Halimi S. & Verges B. Adverse effects and safety of SGLT-2 inhibitors. Diabetes & Metabolism 40, S28–S34 (2014). 25554069 10.1016/S1262-3636(14)72693-X

[b48] Anderson S. L. Dapagliflozin efficacy and safety: a perspective review. Ther. Adv. Drug Saf. 5, 242–254 (2014). 25436106 10.1177/2042098614551938 PMC4232499

[b49] R-Core-Team. R: A language and environment for statistical computing. *R Foundation for Statistical Computing*, *Vienna, Austria*. URL http://www.R-project.org (2013).

[b50] Pinheiro J., Bates D., Debroy S., Deepayan S. & Team, R. D. C. nlme: Linear and Nonlinear Mixed Effects Rodels. R package version 3.1-111 (2013).

