# Supplementary material for: Dapagliflozin stimulates glucagon secretion at high glucose: experiments and mathematical simulations of human A-cells
Source: Sci Rep. 2016 Aug 18;6:31214. doi: 10.1038/srep31214 (PMC4989223; doi:10.1038/srep31214)
Supplement: Supplementary Information [file srep31214-s1.pdf]

Dapagliflozin stimulates glucagon secretion at high glucose: experiments and mathematical simulations of human A-cells

Morten Gram Pedersen #, Ingela Ahlstedt ¤, Michael El Hachmane¤, Sven O. Göpel¤

Supplementary information

Mathematical model equations and parameters

For completeness we report all equations and parameters here.

A mathematical Hodgkin-Huxley-type model that describes electrical activity in human pancreatic A-cells was developed based on patch clamp data from human pancreatic A-cells (1). The model includes ATP-sensitive K^+ channels (KATP-channels), a passive leak current, voltage-gated Na^+-, K^+- and Ca^{2+}-channels, and the electrogenic sodium glucose co-transporter SGLT2.

The evolution of the membrane potential V is driven by the contribution from the different currents,

$$dV/dt = -(I_{KATP} + I_{leak} + I_{Na} + I_{Kv} + I_{KA} + I_{CaT} + I_{CaL} + I_{CaPQ} + I_{SGLT2})/C_m, \quad (1)$$

where C_m =3.3 pF is the cell membrane capacitance. Voltage-gated membrane currents are modeled as

$$I_X = g_X\, m_X^{nX}\, h_X\, (V - V_X), \quad (2)$$

where X stands for the channel type, V_X is the associated reversal potential, g_X the maximal whole-cell channel conductance, and m_X and h_X describe activation and inactivation of the channel, respectively.

Activation (similarly inactivation) is described by

$$dm/dt = (m_{X,\infty}(V) - m)/\tau_{mX} \quad , \quad (3)$$

where $m_{X,\infty}(V)$ is the steady-state voltage-dependent activation function, and τ_{mX} is the time-constant of activation, which in some cases depends on the membrane potential.

Steady-state voltage-dependent activation (inactivation) functions were described by the Boltzmann equation

$$m_{X,\infty}(V) = [1+\exp((V-V_{mX})/S_{mX})]^{-1} . \quad (4)$$

SGLT2 was modeled as a six-state model as described below.

ATP-sensitive K^+ and leak currents

The KATP current was modeled as a linear, passive current

$$I_{KATP} = g_{KATP} (V-V_K) \quad (5)$$

with low conductance g_{KATP}=0.15 nS (1). The K^+ reversal potential was set to V_K=-75 mV. Similarly, the leak current was modeled as

$$I_{leak} = g_{leak} (V-V_{leak}) \quad (6)$$

with conductance g_{leak}=0.1 nS and reversal potential V_{leak}=-20 mV.

Voltage-sensitive Na^+ current

The voltage sensitive Na^+ current was modeled as in the original Hodgkin-Huxley model

$$I_{Na} = g_{Na}\, m_{Na}^3\, h_{Na} (V-V_{Na}) \quad (7)$$

with activation m_{Na} and inactivation h_{Na} described as in Eqs. (3) and (4). Activation was fast with time constant τ_{mNa}=0.05 ms, while inactivation was slower and with voltage-dependent time constant

$$\tau_{hNa} = 0.5 + 60\, [\exp((V+50)/8)+\exp((-V-50)/8)]^{-1} \text{ ms.} \quad (8)$$

Simulated and experimental Na^+-current traces with corresponding IV-curve are shown in Fig. S1. The model tightly reproduces the voltage dependence of the peak currents. The parameters of the steady-state activation function $m_{Na,\infty}(V)$ were V_{mNa}=-25 mV and S_{mNa}=-12 mV. For voltage-gated inactivation published parameters were used (1) for the inactivation function $h_{Na,\infty}(V)$: V_{hNa}=-40 mV, S_{hNa}=5 mV. In order to obtain action potentials in the whole cell model simulated currents needed to be increased compared to published data. The whole-cell conductance was set to g_{Na}=1.6 nS, and the reversal potential was V_{Na}=70 mV.

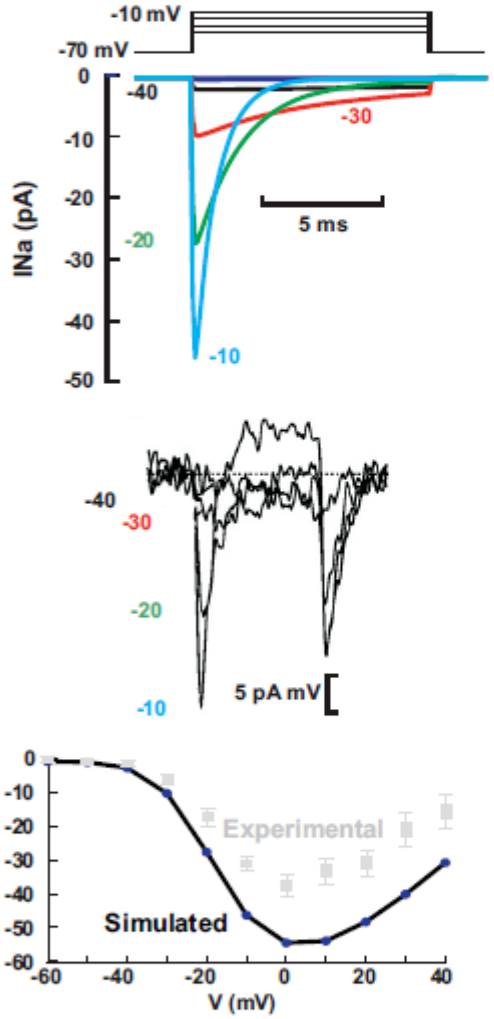

Fig. SI 1: Simulated and experimental Na^{+}-currents. Simulated **(A)** and experimental **(B)** current traces in response to voltage pulses as indicated. IV-curve **(C)** for current traces shown in (A) and (B). Experimental currents are adopted from (1).

Voltage-sensitive K^+ currents

Voltage gated K^+ currents in human A-cells consist of 2 pharmacologically and kinetically separable components. The Kv2.x channel blocker stromatoxin inhibits the slow delayed-rectifier component revealing a fast inactivating A-type current sensitive to Kv4.x channel blocker heteropodatoxin-2 (1). The delayed-rectifier current was modeled as

$$I_{Kv} = g_{Kv}\, m_{Kv}^{4}\, h_{Kv}\, (V-V_K), \qquad (9)$$

with voltage-dependent activation and inactivation,

$$\tau_{mKv} = 4 + 1000\, [0.03 \exp((v+95)/11) + 99 \exp((-v-40)/11)]^{-1} \text{ ms}, \qquad (10)$$

$$\tau_{hKv} = 530 + 4800\, [\exp((v+70)/20)+\exp((-v-70)/20)]^{-1} \text{ ms}. \qquad (11)$$

Conductance and activation parameters were set to tightly reproduce published data (Fig. S2; g_{Kv}=6 nS, V_{mKv}=-10 mV, S_{mKv}=-10 mV), whereas inactivation parameters were taken from Ref. (1): V_{hKv}=-37 mV, S_{hKv}=5 mV.

A-type currents were modeled as (1)

$$I_{KA} = g_{KA}\, m_{KA}^{4}\, h_{KA}\, (V-V_K), \qquad (12)$$

with fast activation τ_{mKA} = 0.18 mS and voltage-dependent inactivation

$$\tau_{hKA} = 10 + 60\, [\exp((v+70)/20)+\exp((-v-70)/20)]^{-1} \text{ ms}. \qquad (13)$$

As for I_{Kv}, conductance and activation were set to tightly reproduce published data (Fig. S2; g_{KA}=9 nS, V_{mKA}=-23 mV, S_{mKv}=-12 mV), whereas inactivation parameters were taken from Ref. (1): V_{hKA}=-49 mV, S_{hKv}=4.8 mV.

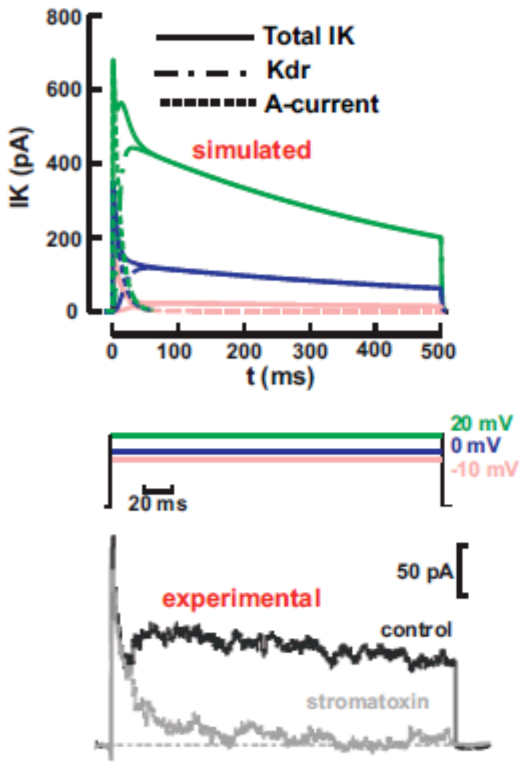

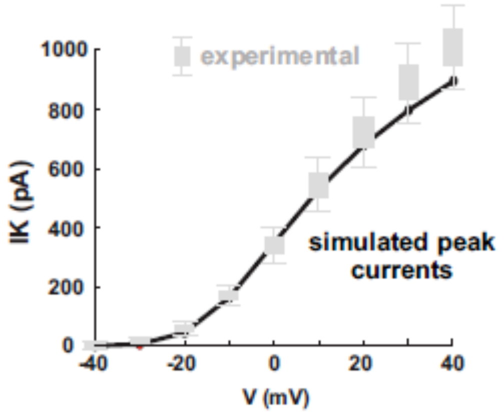

Fig. SI 2: Voltage gated K$^+$-currents. (A) Simulated current traces in response to voltage pulses at different voltages as indicated **B:** Current trace in response to depolarization from -70 to zero mV**.** IV-curve **(C)** for current traces shown in (A) and (B). Experimental currents are adopted from (1).

Voltage-sensitive Ca^{2+} currents

Human A-cells contain at least three types of Ca^{2+} channels: low-voltage-activated T-type channels, and high-voltage-activated L- and P/Q-type channels (1).

The three types of Ca^{2+} currents were modeled as

$$I_{CaT} = g_{CaT}\, m_{CaT}\, h_{CaT}\, (V-V_{Ca}), \quad (14)$$

$$I_{CaL} = g_{CaL}\, m_{CaL}\, h_{CaL}\, (V-V_{Ca}), \quad (15)$$

$$I_{CaPQ} = g_{CaPQ}\, m_{CaPQ}\, h_{CaPQ}\, (V-V_{Ca}). \quad (16)$$

All three currents were assumed to activate rapidly ($\tau_{mCaT} = \tau_{mCaL} = \tau_{mCaPQ} = 0.1$ ms), whereas inactivation occurred on different timescales ($\tau_{hCaT} = 7$ ms, $\tau_{hCaL} = 20$ ms, $\tau_{hCaPQ} = 1000$ ms). Steady-state activation and inactivation functions for T- and L-type Ca^{2+} channels were described as in Eq. (4). The L-type Ca2+ currents in human A-cells show two distinct parts (1). We assumed therefore that the steady-state activation function for L-type Ca^{2+} channels was described as a sum of two Boltzmann functions,

$$m_{CaL,\infty}(V) = 0.1\, [1+\exp((V-V_{mCaL1})/S_{mCaL})]^{-1} + 0.9\, [1+\exp((V-V_{mCaL2})/S_{mCaL})]^{-1}. \quad (17)$$

Inactivation was assumed to be identical for the two subpopulations of L-type Ca^{2+} channels, and was thus described as in Eq. (4). All inactivation parameters were taken from (1), assuming that L- and P/Q-type Ca^{2+} channels inactivation showed similar voltage-dependence, but different kinetics. This assumption was made because of the poor characterization of P/Q-type Ca^{2+} current inactivation. Inactivation parameters were: $V_{hCaT} = -71$ mV, $S_{hCaT} = 8$ mV, $V_{hCaL} = V_{hCaPQ} = -28$ mV, $S_{hCaL} = S_{hCaPQ} = 5$ mV. Conductances and activation parameters were chosen to reproduce experimental data, as discussed below. The parameter values are: $g_{CaT} = 2$ nS, $V_{hCaT} = -40$ mV, $S_{hCaT} = -4$ mV; g_{CaPQ}=1.2 nS, V_{hCaPQ} =-10 mV, $S_{hCaPQ} = -7$ mV; g_{CaL}=1 nS, V_{hCaL1} =-25 mV; $V_{hCaL2} = 0$ mV; S_{hCaL} =-3 mV.

Experimentally obtained Ca^{2+}-currents in response to voltage ramps adopted from (1) and corresponding simulated currents are depicted in Fig. SI 3 upper left. Experimental traces are scaled to the simulated currents in order to facilitate comparison of the voltage dependence. The simulated currents are fairly similar to the experimental data with the most striking difference being the absence of the deflection in the IV-curve between -40 and -20 mV in the experimental data (Fig. SI 3 lower left). Since it is difficult to separate each current component in each individual experiment, the experimental data used to characterize the different current components are partly derived from different experiments. Thus adding up the published data for the individual currents will not yield the total current shown in Ref. (1), Fig. 5B. In our model we aimed at reflecting all published currents and apparently the T-type currents are underrepresented in data from which the IV-curve for the total Ca^{2+}-current was derived explaining the lack of deflection in the experimental IV-curve. Ca^{2+}-currents also often become spontaneously smaller during the experiment, a phenomenon known as “run down” and we thus choose slightly larger currents in our simulations. Simulated and experimental current traces in response to square pulses are shown in Fig. SI 3 right.

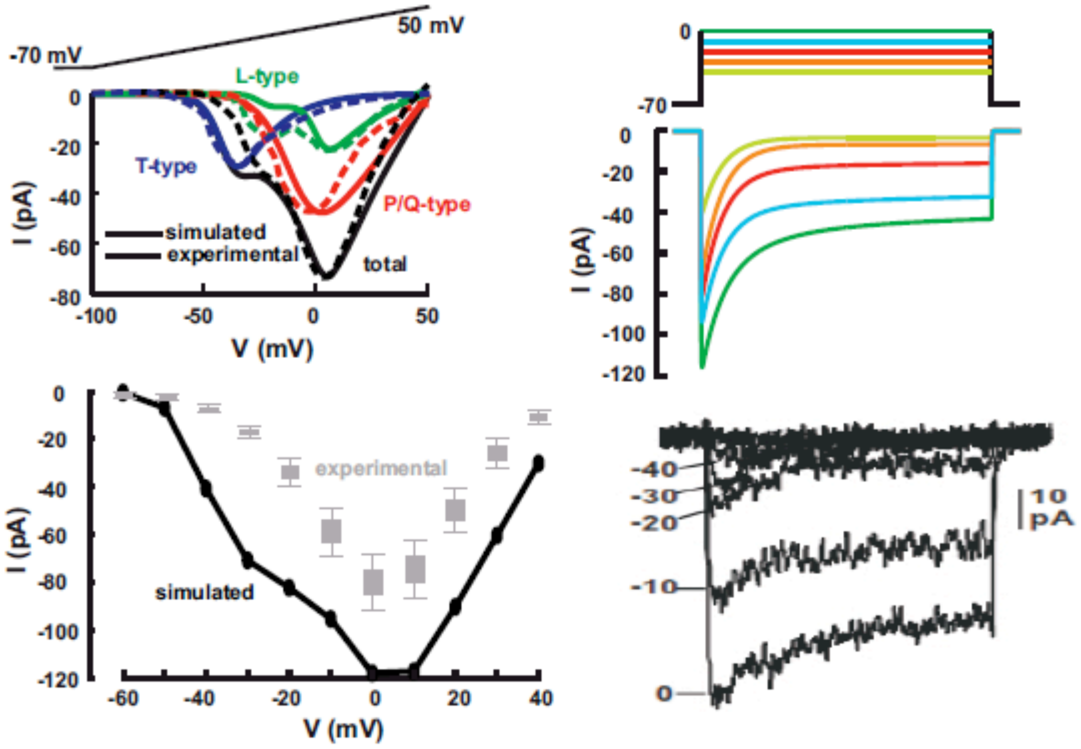

Fig. SI 3: Simulated and experimental Ca^{2+}-currents. A: Current responses upon voltage ramps from -70 to +50 mV for the different Ca^{2+}-channels included in the model and total Ca^{2+}-current as indicated. Experimental curves are scaled to simulated currents. **B:** IV-curve in response to square voltage pulses of simulated and experimental total Ca^{2+}-currents as indicated. **C**: Simulated and experimental currents in response to square pulses to indicated voltages. Experimental currents are adopted from (1).

SGLT2-mediated current

The sodium/glucose co-transporter 2 (SGLT2) utilizes a concentration gradient of Na^+ to transport glucose into the A-cells. Mechanistically SGLT1 and SGLT2 work similarly, with the main divergence being the different stoichometry: SGLT2 transports only one Na^+ ion, whereas SGLT1 transport two, for each molecule of glucose transported into the cell. Moreover, in absence of sodium in the external medium, glucose is not transported (2).

Parent et al. (3) developed a six-state model for Na^+/glucose co-transport by SGLT1, which we used as starting point for the SGLT2 model used here. Rate expressions and constants were modified to account for species differences (4), and differences between SGLT1 and SGLT2 (5).

The model is depicted in Fig. SI 4. Starting with the empty carrier outside the cell (state 1), the first step is the association of a sodium ion with the carrier (state 2), which allows the subsequent association of glucose (state 3). The third step corresponds to the translocation of the carrier from outside to inside the cell (state 4). Symmetrical steps take place inside the cell consisting in successive dissociation of glucose (state 5) and sodium (state 6). A final step brings the empty carrier back to the initial state outside the cell.

The model equations describe translocation between the different states of the transporter,

$$dC_1/dt = (k_{21}C_2 + k_{61} C_6) - (k_{12} + k_{16}) C_1 , \quad (18)$$

$$dC_2/dt = (k_{12}C_1 + k_{32} C_3 + k_{52} C_5) - (k_{21} + k_{23} + k_{25}) C_2 , \quad (19)$$

$$dC_3/dt = (k_{23}C_2 + k_{43} C_4) - (k_{32} + k_{34}) C_3 , \quad (20)$$

$$dC_4/dt = (k_{34}C_3 + k_{54} C_5) - (k_{45} + k_{43}) C_4 , \quad (21)$$

$$dC_5/dt = (k_{45}C_4 + k_{65} C_6 + k_{25} C_2) - (k_{54} + k_{52} + k_{56}) C_5 , \quad (22)$$

$$dC_6/dt = (k_{16}C_1 + k_{56} C_5) - (k_{61} + k_{65}) C_6 , \quad (23)$$

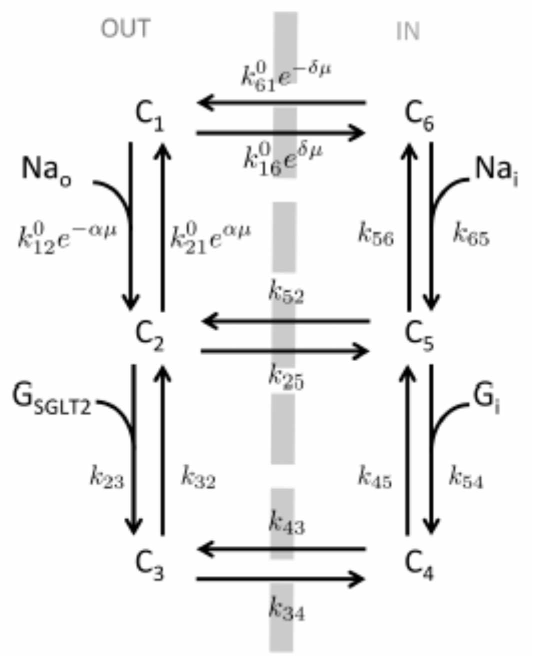

Fig. SI 4: Six-state model of SGLT2. The model is based on Parent et al. (3) and Mackenzie et al. (5). Parameters are unless otherwise mentioned: Na_i=0.2 mM, Na_o=140 mM, G_i=0.1 mM, G_{SGLT2}=11 mM, α=0.3, k_{12}^0=5e-05 mM^{-1}, k_{21}^0=0.3, k_{23}^0=0.045 mM^{-1}, k_{32}=0.02, k_{25}=1e-05, k_{52}=2.2e-05, k_{34}=0.05, k_{43}=0.05, k_{45}=0.8, k_{54}^0=4 mM^{-1}, k_{56}=0.016, k_{65}^0 =5e-08 mM^{-1}, δ=0.7, k_{16}^0=0.6, k_{61}^0 =0.025.

where

$$k_{12}=k_{12}^0\, Na_o \exp(-\alpha\mu)\ ,\ k_{21}=k_{21}^0 \exp(\alpha\mu)\ ,\ k_{23}=k_{23}^0\, G_{SGLT2}\ ,\ k_{54}=k_{54}^0\, G_{SGLT2}\ , \qquad (24)$$

$$k_{65}=k_{65}^0\, Na_i\ ,\ k_{16}=k_{16}^0 \exp(\delta\mu)\ ,\ k_{61}=k_{61}^0 \exp(-\delta\mu).$$

A small current is associated with sodium/glucose co-transport, attributable to the translocation of the negatively charged carrier (3),

$$I_{SGLT2} = -\,(F\, n/N_A)\ [\alpha(k_{12}C_1 - k_{21}C_2)+\delta(k_{61}C_6 - k_{16}C_1)], \qquad (18)$$

where F is the Faraday constant, n the number of transporters, N_A the Avogadro's number, k_{xy} is the rate constant describing the transition between state x and state y, C_z is the fraction of carriers in state z, and α and δ are phenomenological coefficients representing fractional dielectric distances. Finally, μ is the reduced potential F V/RT, where R is the gas constant and T is the temperature. The SGLT2 current depends on glucose and sodium concentrations inside and outside the cell, as well as on the membrane voltage V, because of the dependence of the rate constants on these factors.

The magnitude of the SGLT2 current is directly proportional to the number of transporters n in the cell. We use n=1.5 x 10^9. The number of SGLT2 transporters is constrained by the electrophysiology. Increasing n 5 or 10 fold disturbs simulated activity, which no longer appears similar to experimental recordings (Fig. SI 5)

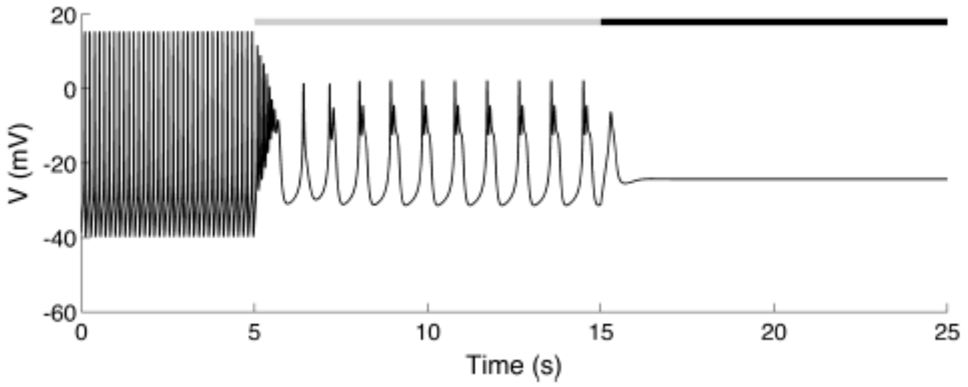

Fig. SI 5: Electrophysiological properties constrain the number of SGLT2 transporters. Simulated electrical activity with default parameter n=1.5 x 10^9, with a 5-fold increase (n=7.5 x 10^9, grey bar), and with 10-fold increase (n=15 x 10^9, black bar).

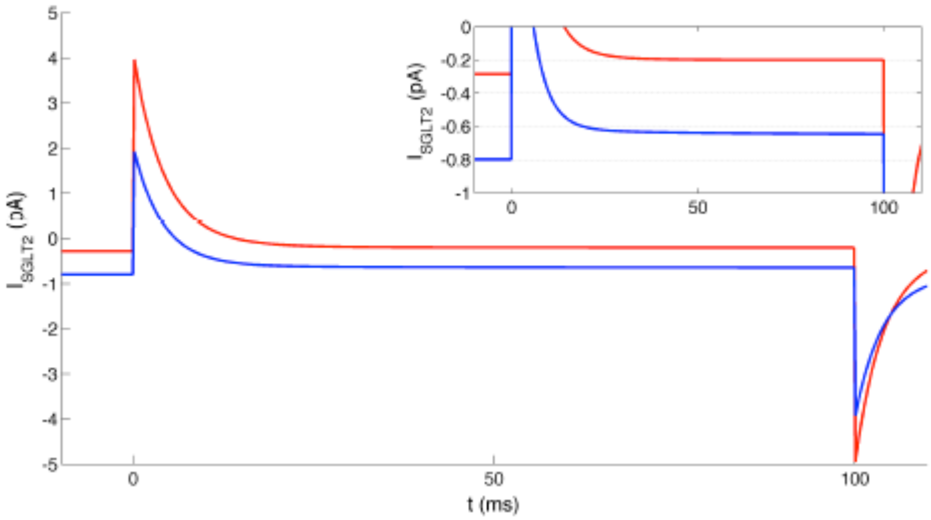

Figure SI 6. **Simulated SGLT2 currents during depolarizations from -70 to -60 mV in 1 mM (red) or 11 mM (blue) glucose.** The calculated conductances are, respectively, (-0.200 – (-0.284))pA/10 mV = 8.4 pS and (-0.646 – (-0.799)) pA/10 mV = 15.3 pS. Thus, 11 mM glucose increases the part of the background conductance due to SGLT2 by ~7 pS compared to 1 mM, which will mask small glucose-induced decreases in the KATP conductance.

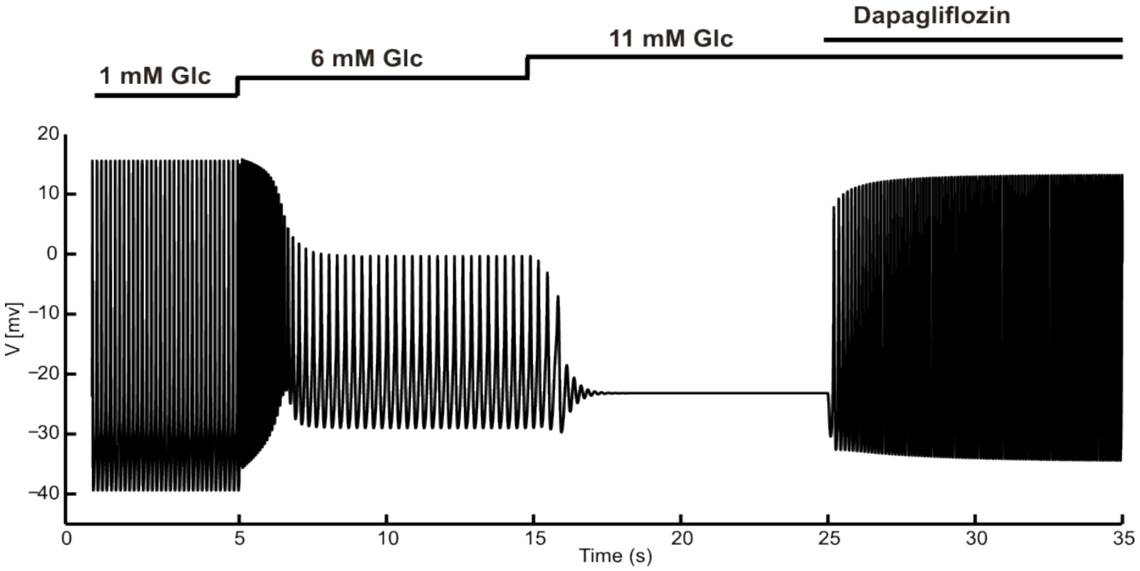

Figure SI 7. **Higher SGLT2 density leads to more pronounced effects in the model.** Simulated membrane voltage as in Fig. 2, but with 10% more SGLT2 transporters ($n=1.65 \times 10^9$), shows reduced AP height at 6 mM glucose, and absence of AP firing at 11 mM glucose. Dapagliflozin application restores AP firing.

Supplementary References

1. Ramracheya R*, et al.* (2010) Membrane Potential-Dependent Inactivation of Voltage-Gated Ion Channels in α-Cells Inhibits Glucagon Secretion From Human Islets. *Diabetes* 59(9):2198-2208.

2. Wright EM, Loo DDF, & Hirayama BA (2011) Biology of human sodium glucose transporters. *Physiol. Rev.* 91:733-794.

3. Parent L, Supplisson S, Loo D, & Wright EM (1992) Electrogenic properties of the cloned Na+/glucose cotransporter: I. Voltage-clamp studies. *J. Membr. Biol.* 125:49-62.

4. Hirayama BA*, et al.* (1996) Kinetic and specificity differences between rat, human, and rabbit Na+-gllucose cotransporters (SGLT-1). *Am. J. Physiol.* 270:G919-926.

5. Mackenzie B, Loo DDF, Panayotova-Heiermann M, & Wright EM (1996) Biophysical characteristics of the pig kidney Na+/glucose cotransporter SGLT2 reveal a common mechanism for SGLT1 and SGLT2. *J. Biol. Chem.* 271(51):32678-32683.
